# Retraction Note: An apoptosis-enhancing drug overcomes platinum resistance in a tumour-initiating subpopulation of ovarian cancer

**DOI:** 10.1038/s41467-020-15721-y

**Published:** 2020-04-30

**Authors:** D. M. Janzen, E. Tiourin, J. A. Salehi, D. Y. Paik, J. Lu, M. Pellegrini, S. Memarzadeh

**Affiliations:** 1 Department of Obstetrics and Gynecology, David Geffen School of Medicine, University of California, Los Angeles, Los Angeles, CA 90095 USA; 2 Department of Molecular, Cell and Developmental Biology, University of California, Los Angeles, Los Angeles, CA 90095 USA; 3 Eli and Edythe Broad Center of Regenerative Medicine and Stem Cell Research, University of California, Los Angeles, Los Angeles, CA 90095 USA; 4 The VA Greater Los Angeles Health Care System, Los Angeles, CA 90073 USA

Retraction of: *Nature Communications* 10.1038/ncomms8956, published online 3 August 2015

An investigation of archival materials initiated by the corresponding author of this paper revealed several errors that undermine the central conclusions with regards to the sensitivity of ovarian cancer tumours to the co-therapy of birinapant and carboplatin in vivo. STR profiling evaluation of archival material pertaining to several key experiments revealed that Ovcar-3 cells were used in xenografts rather than as stated, S1-GODL cells in Figs. 6b, c, 7 and Supplementary Fig. 8, patient sample II cells in Fig. 8, and MCF7 cells in Supplementary Fig. 9. Given these issues, we wish to retract the Article in its entirety. We deeply regret this circumstance and apologize to the community.

